# Association between tea consumption and risk of cognitive disorders: A dose-response meta-analysis of observational studies

**DOI:** 10.18632/oncotarget.17429

**Published:** 2017-04-26

**Authors:** Xueying Liu, Xiaoyuan Du, Guanying Han, Wenyuan Gao

**Affiliations:** ^1^ School of Pharmaceutical Science and Technology, Tianjin University, Nankai District, Tianjin 300072, China; ^2^ Department of Pharmacy, The Third Affiliated Hospital of Jinzhou Medical University, Jinzhou 121001, China; ^3^ Department of Pathology, The First Affiliated Hospital of Jinzhou Medical University, Jinzhou 121001, China; ^4^ Department of Pharmacy, The First Affiliated Hospital of Jinzhou Medical University, Jinzhou 121001, China

**Keywords:** tea consumption, cognitive disorders, dose-response, meta-analysis

## Abstract

**Background:**

The epidemiological evidence for a dose-response relationship between tea consumption and risk of cognitive disorders is sparse. The aim of the study was to summarize the evidence for the association of tea consumption with risk of cognitive disorders and assess the dose-response relationship.

**Methods:**

We searched electronic databases of Pubmed, Embase, and Cochrane Library (from 1965 to Jan 19, 2017) for eligible studies that published in the international journals. A random-effects model was used to pool the most adjusted odds ratios (ORs) and the corresponding 95% confidence intervals (CIs).

**Results:**

Seventeen studies involving 48,435 participants were included in our study. The meta-analysis showed that a higher tea consumption was associated with a significant reduction in the risk of cognitive disorders (OR=0.73, 95% CI: 0.65-0.82). When considering the specific types of tea consumption, the significantly inverse association is only found in green tea consumption (OR=0.64, 95% CI: 0.53-0.77) but not in black/oolong tea consumption (OR=0.75, 95% CI: 0.55-1.01). Dose-response meta-analysis indicated that tea consumption is linearly associated with a reduced risk of cognitive disorders. An increment of 100 ml/day, 300 ml/day, and 500 ml/day of tea consumption was associated with a 6% (OR=0.94, 95% CI: 0.92-0.96), 19% (OR=0.81, 95% CI: 0.74-0.88), and 29% (OR=0.71, 95% CI: 0.62-0.82) lower risk of cognitive disorders.

**Conclusions:**

Tea consumption is inversely and linearly related to the risk of cognitive disorders. More studies are needed to further confirm our findings.

## INTRODUCTION

In the rapidly aging societies around the world, cognition-related diseases, such as mild cognitive impairment, cognitive decline, dementia and Alzheimer's disease (AD), are gradually increasing [1, 2]. The number of people with dementia worldwide is about 35.6 million as announced by WHO and this number will be doubled by 2030, tripled by 2050 [3]. Interventions against early cognitive disorders may be particularly effective to reduce the social burden of AD and other types of dementia. It has been indicated that the intake of certain diet and nutrients, such as fruit and vegetable [4], Mediterranean diet [5], omega-3 fatty acids [6], vitamin C [7], vitamin E [8], milk [9], coffee [10], and light to moderate amounts of alcohol [11], is related to the reduced risk of cognitive disorders and dementia.

A number of epidemiological studies have found that tea consumption may also improve mental performance [12] and reduce the progression of cognitive dysfunction [13, 14]. An animal study has shown that oolong and green teas could reduce the deteriorations of cognitive ability, brain degenerative changes and aging process in senescence accelerated-prone mice P8 [15]. Experimental studies indicated that the anti-oxidative and anti-inflammatory effects of tea and its components, such as catechins and theanine, may contribute to neuroprotection [16–20].

The strength of the association between tea consumption and the risk of cognitive disorders remains uncertain due to the differences in participants and methodological methods used in the previous studies. In the current study, we conducted a systematic review and dose-response meta-analysis to quantitatively assess the association between tea consumption and the risk of cognitive disorders.

## RESULTS

### Description of the included studies

**Figure 1:**
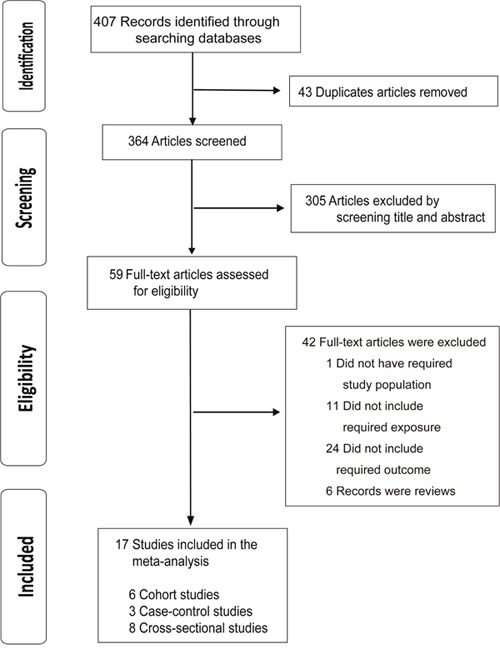
Flowchart for the selection of eligible studies

Of the 407 citations identified from the database searches, 17 studies met the inclusion criteria and were finally included in the meta-analysis, including six cohort studies [21–26], three case-control studies [27–29] and eight cross-sectional studies [3, 30–36]. The study selection process is shown in Figure 1. Among the included studies, twelve were from Asia, two were from Europe, two were from North America, and one was from Australia, involving a total of 48,435 participants. The characteristics of these studies are summarized in Table 1. All included studies measured tea consumption by self-administered questionnaire or self-reported food frequency questionnaire. The diagnosis of cognitive disorders was based on standard criterion in all the articles. Most of the included studies adjusted for potential confounding factors. We recorded relative risks of cognitive disorders according to the highest vs. the lowest category of tea consumption. The results of quality evaluation for each study are presented in detail in the Supplementary Materials (Supplementary Tables 3–5).

**Table 1:** Characteristics of included studies in the systematic review and meta-analysis

Study	Country	Study design	Sample size	Mean age (years)	Follow-up duration (years)	Exposure variable	Disease type	Disease ascertainment	Adjustment
Broe et al, 1990	Australia	Case-control	340	77.5	-	All tea	AD	NINCDS-ADRDA	Age, sex and the general practice of origin
Chen et al, 2012	China	Prospective nested case-control study	5691	89.2	3	All tea	Cognitive decline	MMSE	None
Dai et al, 2006	The United States	Cohort	1589	71.8	6.4	All tea	AD	NINCDS-ADRDA	Years of education, gender, regular physical activity, BMI, baseline CASI score, olfaction diagnostic group, total energy intake, intake of saturated, monounsaturated, and polyunsaturated fatty acids, ApoE genotype, smoking status, alcohol drinking, supplementation of vitamin C, vitamin E, and multivitamin, tea drinking, fruit and vegetable juice drinking, dietary intake of vitamin C, vitamin E, and β-carotene.
Eskelinen et al, 2009	Finland	Cohort	1409	50.2	21	All tea	Dementia, AD	MMSE, DSM-IV and NINCDS-ADRDA	Age, sex, education, follow-up time, community of residence, midlife smoking, systolic blood pressure, serum total cholesterol, BMI, and physical activity.
Forster et al, 1995	The United Kingdom	Case-control	218	55.9	-	All tea	AD	NINCDS-ADRDA	None.
Huang et al, 2009	China	Cross-sectional	681	93.5	-	All tea	Cognitive impairment	MMSE	Age, sex, sleep habits, educational level, religion habits, and temperament.
Kitamura et al, 2016	Japan	Cross-sectional	1143	68.9	-	All tea; green tea	Cognitive impairment	MMSE	Age, BMI, history of stroke, history of myocardial infarction, walking time, alcohol intake, and fruit consumption.
Kuriyama et al, 2006	Japan	Cross-sectional	1003	74.7	-	All tea and green tea	Cognitive impairment	MMSE	Age, sex, energy intake, intake of nondietary vitamin C or E, fish consumption, green or yellow vegetable consumption, mild leisure-time physical activity, vigorous leisure-time physical activity, smoking, and alcohol use.
Lindsay et al, 2002	Canada	Cohort	4088	73.3	5	All tea	AD	DSM-IV	Age, sex, and education.
Ng et al, 2008	Singapore	Cross-sectional	2607	66	-	All tea; green tea; black and oolong tea	Cognitive impairment, cognitive decline	MMSE	Age, sex, education, smoking, alcohol consumption, BMI, hypertension, diabetes, heart disease, stroke, depression, APOEε4, physical activities, social and productive activities, vegetable and fruit consumption, fish consumption, and coffee consumption.
Noguchi-Shinohara et al, 2014	Japan	Cohort	490	71.2	4.9	Green tea; black tea	Dementia, cognitive decline	MMSE, CDR and DSM-III-R	Age, sex, history of hypertension, diabetes mellitus, typerlipidemia, education, APOE ε4 carrier status, alcohol drinking, smoking, physical activities and/or hobbies, and coffee consumption.
Shen et al, 2015	China	Cross-sectional	9375	70	-	All tea; green tea	Cognitive impairment	CCM and MMSE	Age, sex, race, education, marriage, tea concentration, tea categories, physical examinations, family status, disease situation, behavioral risk factors, dietary intake, nutrition supplement, depression and ADL.
Tomata et al, 2016	Japan	Cohort	13645	73.8	5.7	Green tea; black tea; oolong tea	Dementia	LTCI system and cognitive function score	Age, sex, history of disease, educational level, smoking, alcohol drinking, BMI, psychological distress score, time spent walking, social support, participation in community activities, motor function score, consumption volume of specific foods coffee consumption, and energy intake.
Wang et al, 2014	China	Cohort	223	70.9	2	Green tea	Cognitive decline	MMSE	Age and gender.
Wang et al. 2016	China	Cross-sectional	1005	72.7	-	All tea	Cognitive impairment	Clinical diagnosis and MMSE	Not mention.
Wu et al, 2011	Taiwan	Cross-sectional	2119	73.3	-	All tea	Cognitive impairment	MMSE	Age, gender, educational level, marital status, social support, hyperlipidemia, stroke, physical function, depressive symptoms, self-rated health, cigarette smoking, leisure-time physical activity, fruits and vegetables consumption, coffee intake, multivitamin intake, and BMI.
Yao et al, 2010	China	Cross-sectional	2809	70.6	-	All tea	Cognitive impairment	MMSE	None.

*Abbreviations*: AD, Alzheimer's disease; ADL, Activities of Daily Living; BMI: body mass index; CASI, Cognitive Abilities Screening Instrument; CDR, Clinical Dementia Rating Scale; DSM-III-R, Diagnostic and Statistical Manual of Mental Disorders, Third Edition, Revised; DSM-IV, Diagnostic and Statistical Manual of Mental Disorders, Fourth Edition; LTCI, Long-term Care Insurance; MMSE, Mini-Mental State Examination; NINCDS-ADRDA, National Institute of Neurological and Communicative Diseases and Stroke-Alzheimer Disease and Related Disorders Association.

### All types of tea consumption and risk of cognitive disorders

**Figure 2:**
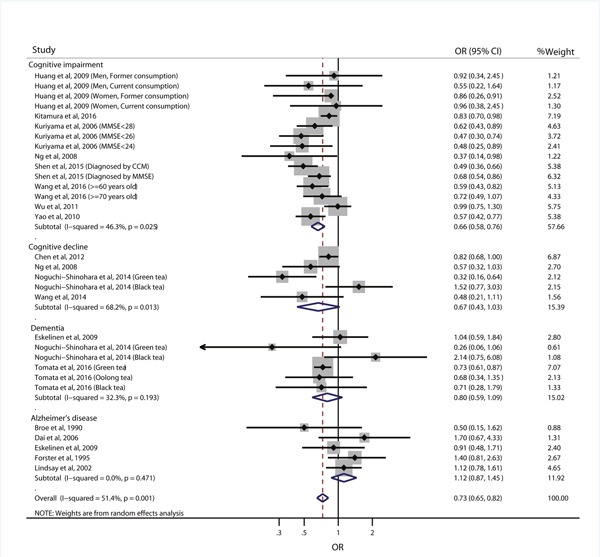
Relative risk of cognitive disorders according to the highest vs. lowest category of all types of tea consumption

Figure 2 shows the results of meta-analysis of relative risk according to the highest vs. lowest category of all types of tea consumption. The summary result showed that high tea consumption was associated with a reduced risk of cognitive disorders (OR=0.73, 95% CI: 0.65-0.82), with some heterogeneity (*I*^2^=51.4%, *p*=0.001). In the stratified analysis by the type of outcome, the pooled ORs and 95% CIs of higher tea consumption were 0.66 (0.58-0.76) for cognitive impairment, 0.67 (0.43-1.03) for cognitive decline, 0.80 (0.59-1.09) for dementia, and 1.12 (0.87-1.45) for Alzheimer's disease. Subgroup analyses by study design showed that the inverse association between tea consumption and risk of cognitive disorders was found in both cohort studies (OR=0.84, 95% CI: 0.74-0.95) and cross-sectional studies (OR=0.66, 95% CI: 0.58-0.75) (Table 2). Sensitive analysis showed that the inverse association was not materially changed in the leave-one-out analyses by omitting one study in turn, with pooled ORs range from 0.71 (95% CI: 0.63-0.80) to 0.0.75 (95 CI: 0.66-0.84) (Supplementary Figure 1).

**Table 2:** Results of subgroup analyses by study design

Exposure variable	Study design	Number of estimates	Pooled OR (95% CI), *I*^2^ statistics (%), and *P*-value for the heterogeneity *Q* test
All types of tea consumption	Cohort	12	0.84 (0.74-0.95); *I*^2^=56.6%, *P*=0.01
	Cross-sectional	16	0.66 (0.58-0.75); *I*^2^=43.4%, *P*=0.03
Green tea consumption	Cohort	3	0.46 (0.23-0.95); *I*^2^=70.9%, *P*=0.03
	Cross-sectional	8	0.66 (0.53-0.83); *I*^2^=60.6%, *P*=0.01
Black/oolong tea consumption	Cohort	4	1.08 (0.63-1.84); *I*^2^=41.2%, *P*=0.16
	Cross-sectional	3	0.61 (0.49-0.75); *I*^2^=0.0%, *P*=0.52
An increment of 100 ml/day of tea consumption	Cohort	6	0.96 (0.93-0.99); *I*^2^=34.8%, *P*=0.18
	Cross-sectional	9	0.92 (0.89-0.95); *I*^2^=50.9%, *P*=0.04
An increment of 300 ml/day of tea consumption	Cohort	6	0.91 (0.84-0.99); *I*^2^=63.2%, *P*=0.02
	Cross-sectional	9	0.77 (0.72-0.83); *I*^2^=27.1%, *P*=0.20
An increment of 500 ml/day of tea consumption	Cohort	6	0.84 (0.61-0.98); *I*^2^=67.8%, *P*=0.01
	Cross-sectional	9	0.69 (0.63-0.75); *I*^2^=0.0%, *P*=0.45

Four estimates from two individual studies were available for assessing the relative risk for men and women, separately. The pooled results also showed a trend of reduced risk of cognitive disorders in both men and women by higher tea consumption, but did not reach statistical significances (OR=0.65, 95% CI: 0.38-1.11 and OR=0.63, 95% CI: 0.38-1.07, respectively) (Supplementary Figure 2).

### Different types of tea consumption and risk of cognitive disorders

**Figure 3:**
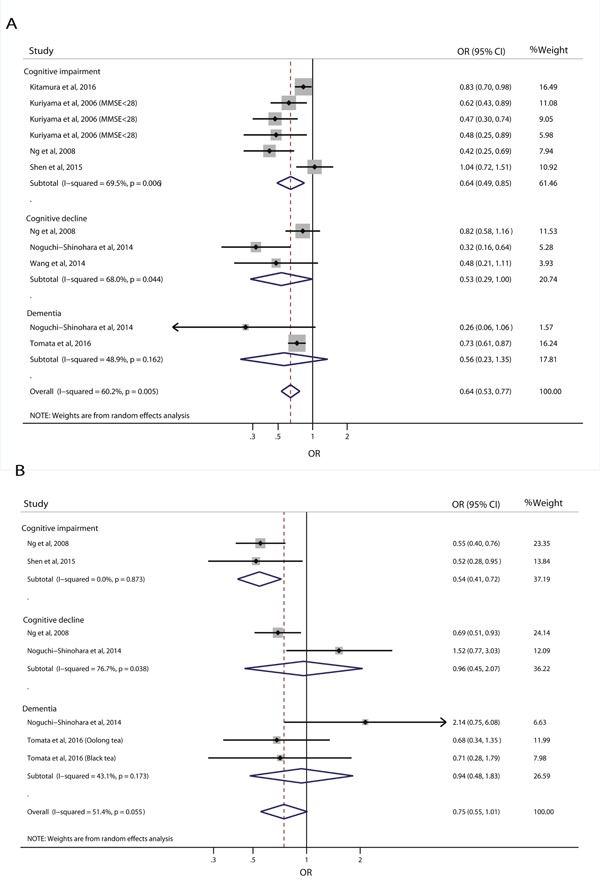
Relative risk of cognitive disorders according to the highest vs. lowest category of green tea **(A)** and black/oolong tea **(B)** consumption.

Eleven estimates from seven individual studies were available for analysis of green tea consumption and the risk of cognitive disorders and seven estimates from four individual studies were available for analysis of black/oolong tea consumption and risk of cognitive disorders. The pooled analyses showed that higher green tea consumption was associated with a reduced risk of cognitive disorders (OR=0.64, 95% CI: 0.53-0.77) (Figure 3A); however, there was no significant association between higher black/oolong tea consumption and risk of cognitive disorders (OR=0.75, 95% CI: 0.55-1.01) (Figure 3B). Subgroup analyses by study design showed that the inverse association between green tea consumption and risk of cognitive disorders was found in both cohort studies (OR=0.46, 95% CI: 0.23-0.95) and cross-sectional studies (OR=0.66, 95% CI: 0.53-0.83); higher black/oolong tea consumption was associated with a reduced risk of cognitive disorders in cross-sectional studies (OR=0.61, 95% CI: 0.49-0.75) (Table 2).

### Dose-response analyses

**Figure 4:**
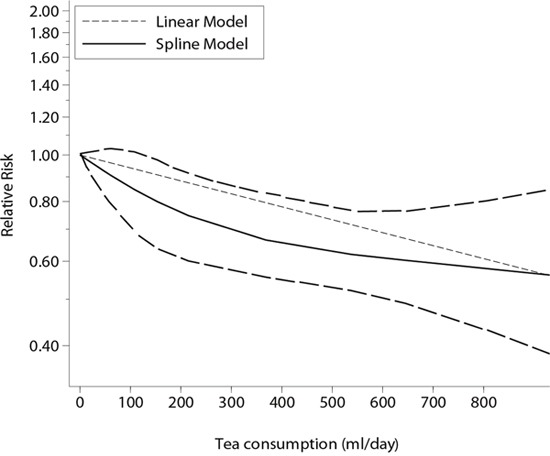
Dose-response relationship between tea consumption and risk of cognitive disorders

**Figure 5:**
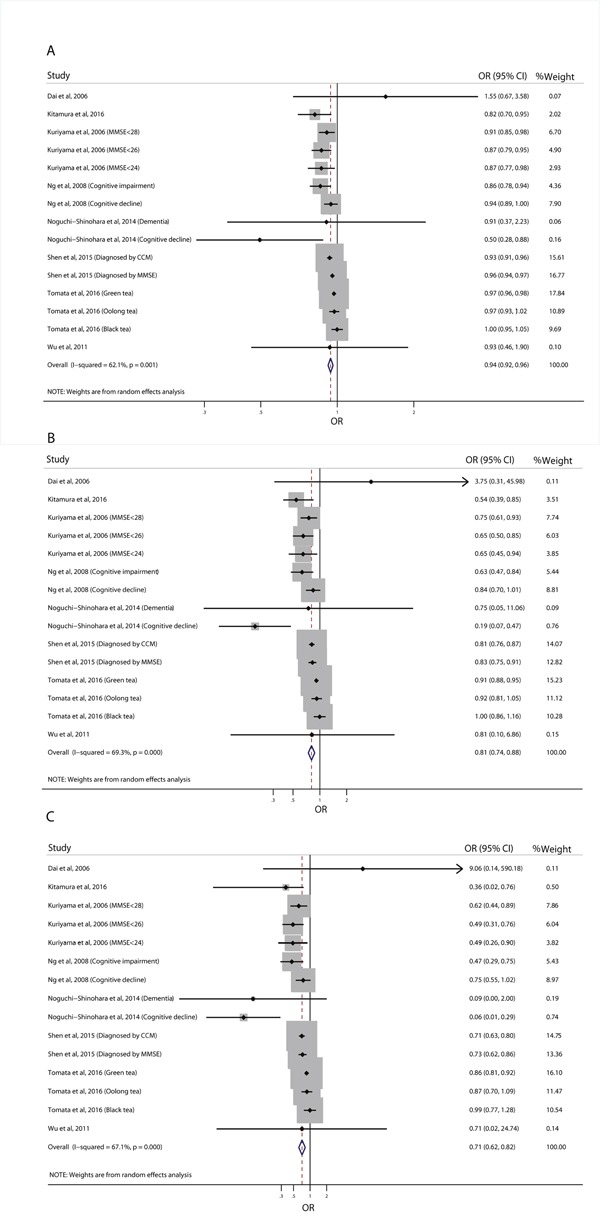
Relative risk of cognitive disorders for an increment of 100 ml/day **(A)**, 300 ml/day **(B)**, and 500 ml/day **(C)** of tea consumption.

Fifteen estimates from eight individual studies were included in the dose-response meta-analyses. Other studies were not included because there were only two categories of tea consumption in these studies, and dose-response meta-analysis requires data for the distribution of cases and person-time across at least three categories of exposure [37]. As shown in Figure 4, there was a linear relationship between tea consumption and risk of cognitive disorders (p for linear trend=0.042; p for non-linear trend=0.236). The dose-response meta-analyses showed that an increment of 100 ml/day, 300 ml/day, and 500 ml/day of tea consumption was associated with a 6% (OR=0.94, 95% CI: 0.92-0.96), 19% (OR=0.81, 95% CI: 0.74-0.88), and 29% (OR=0.71, 95% CI: 0.62-0.82) lower risk of cognitive disorders, respectively (Figure 5). The inverse association was not materially changed when subgrouped by study design (Table 2).

### Publication bias

**Figure 6:**
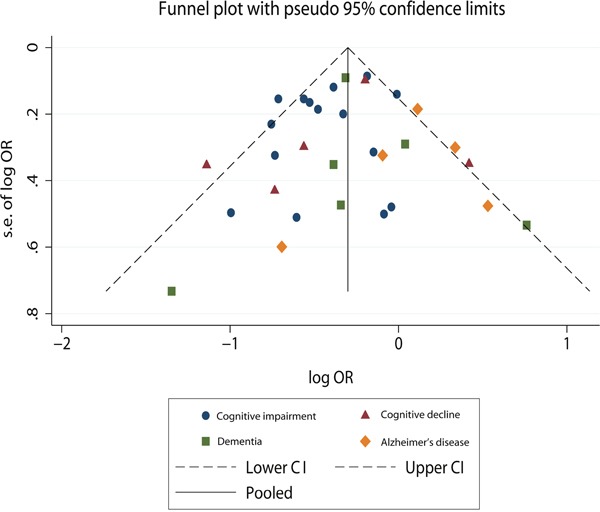
Funnel plot to explore publication bias The vertical line is at the mean effect size.

Visual assessment of funnel plot (Figure 6) showed that the studies were distributed fairly symmetrically about the combined effect size in the meta-analysis of all types of tea consumption and risk of cognitive disorders, which suggests little publication bias. Egger's regression test (*p*=0.678) and Begg-Mazumdar test (*p*=0.747) further confirmed that there was no potential publication bias.

## DISCUSSION

Tea is a commonly consumed beverage not only in Asia but also in other parts of the world and has been reported to have unlimited health benefits. To our knowledge, this study is the first dose-response meta-analysis to evaluate the effect of tea consumption on the risk of cognitive disorders. The findings from our meta-analysis indicated that tea consumption is inversely associated with the risk of cognitive disorders. However, when considering the specific types of tea consumption, the significantly inverse association is only found in green tea consumption but not in black/oolong tea consumption. Overall, the risk decreases by 6%, 19%, and 29% for every 100 ml/day, 300ml/day, and 500ml/day increase in tea consumption, respectively.

A previous meta-analysis from Kim *et al*. [38] found that caffeine intake from coffee or tea was not associated with the risk of cognitive disorders. When subgrouped by caffeine source, caffeine intake from tea also showed no association with cognitive disorders. However, in their study, they used caffeine intake from tea as the exposure variable of interest, while our study used the overall tea consumption as the exposure. Systematic reviews from Arab *et al*. [39] and Panza et al. [40] assessed epidemiologic evidence of the relationship between tea, coffee, or caffeine consumption and cognitive decline. However, the included studies that specifically for tea consumption were rare and pooled analyses were not conducted in these two reviews. In addition, although they found the estimates of cognitive decline were lower among consumers, there is a lack of a distinct dose response. The findings of our study extend the results of a previous cohort study [41], which found that tea consumption is significantly associated with a lower risk of incident functional disability, such as stroke, cognitive impairment, and osteoporosis. In Feng et al's study [14], verbal fluency test was used as measure of cognitive function. This study found that daily and occasional tea drinkers had significantly higher verbal fluency scores compared with non-drinkers, and regular tea drinking was associated with better cognitive function in oldest-old Chinese. In Ide *et al*.'s study [13], they found that green tea consumption was not only effective in improving cognitive function, but also effective in reducing the progression of cognitive dysfunction.

Arab et al.'s study [42] found that some levels of tea consumption modestly reduced rates of cognitive decline among the women but not men. Thus, sex may be an important factor that impacts the association between tea consumption and risk of cognitive disorders. However, there were very limited studies that focused on this association for men and women separately. In the current meta-analysis, only four estimates from two individual studies were available for assessing the relative risks for men and women, separately; and our results showed that neither male nor female tea consumers showed a significant association with the risk of cognitive disorders. This may be due to the very limited studies included in the meta-analyses, which led to low statistical powers. Therefore, more studies are needed to further assess the association between tea consumption and risk of cognitive disorders in difference sexes.

There are several plausible mechanisms to explain the protective effect of tea consumption against cognitive disorders. Many studies have shown that oxidative stress, which occurs by oxidizing macromolecules such as proteins, lipids, and DNA, is an important pathogenic factor in cognitive diseases including AD [43–46]. Tea is enriched in polyphenols and the major tea-related polyphenols present in green tea are catechins, which are polyphenolic compounds with high antioxidant capacities [47], and they have shown effects on anti-aging [48], anti-diabetic [49, 50], anti-stroke [51], and anti-cancer [52] in various studies. Polyphenols contained in green tea have also been reported to have anti-amyloidogenic effects, and could be a key molecule for the development of preventives and therapeutics for AD [53, 54]. Coimbra *et al*.'s study [20] showed that green tea can significantly reduce the serum levels of malonyldialdehyde and malonyldialdehyde+4-hydroxy-2(E)-nonenal, the lipid peroxidation products, and reduce the oxidative stress within the erythrocyte. L-theanine, a free amino acid naturally found in tea, can also play a neuroprotective role [55]. Caffeine, another important component of tea leaf, has been known for its refreshing effect and is beneficial for performance improvement on attention tasks when combined with L-theanine [56, 57]. Chen *et al*. [58] demonstrated that one mechanism implicated in the pathogenesis of AD is blood-brain barrier dysfunction and caffeine exerts protective effects against AD at least in part by keeping the blood-brain barrier intact. Furthermore, previous studies supported the biological effects of caffeine on brain function [59], including modulation of white matter lesions and/or microvascular ischemic lesions [60]. Another potential mechanism for long-term neuroprotective effect of caffeine may involve blockade of adenosine A2A receptors [61], which may attenuate injury caused by β-amyloid, the toxic peptide that accumulates in the brain of patients with AD [62, 63]. In fact, both acute and long-term caffeine intake were shown to suppress β-amyloid levels in plasma and brain of AD transgenic mice [64, 65] and memory restoration and reversal of AD pathology in mice with preexisting β-amyloid burden [66]. In addition, tea consumption has shown to reduce the hypertension risk [67] and decrease the serum concentrations of total cholesterol (TC) and low density lipoprotein cholesterol (LDL-C) [68]; hypertension and dyslipidemia are well known to be the risk factors for atherosclerosis [69, 70], and atherosclerosis is in turn associated with cognitive dysfunction. Thus, tea consumption may reduce the risk of cognitive disorders indirectly by reducing the relevant health problems on atherosclerosis.

In our study, green tea consumption but not in black/oolong tea consumption was associated with reduced risk of cognitive disorders. This may be due to the fact that the nutritional and functional components are not quite the same between green tea and black/oolong tea. For example, the levels of catechin (epigallocatechin gallate [ECGC]) are highest in green tea, followed in order by oolong tea and black tea [71]. Moreover, green tea contains ascorbic acid and high intake of ascorbic acid is related to the reduced risk of AD [72]; however, black tea do not contain ascorbic acid [22]. The differences in the protective effects of cognitive function between green tea and black/oolong tea need to be further clarified by more mechanistic studies.

The strengths of the present meta-analysis include less influence exerted by small-study bias, greatest extent of control for confounding factors, a moderate-to-high quality of studies included in the meta-analysis, and no evidence of publication bias. Moreover, we performed dose-response meta-analysis and found a linear relationship between tea consumption and risk of cognitive disorders.

However, our study has several limitations. First, we found a mild heterogeneity within the studies, which could be explained by the difference in study designs, study populations, measure methods of tea and cognitive disorders, and analytical strategies. Second, the association was evaluated by using the most adjusted model in each included study; however, some important confounding factors, such as lifestyle (diets, hobbies, physical activities, etc.), cultural differences, lifestyle-related diseases (diabetes mellitus, hypertension, dyslipiedemia, etc.) and ApoE status were not adjusted in some of the included studies. In addition, we cannot exclude chance, residual or unmeasured confounding as the alternative explanation of our results. Thus, our results must be explained with caution. Third, in most of the included studies, the consumption of tea was measured using a self-administered questionnaire; and thus, misclassification is inevitable. Fourth, although the overall analysis was based on a large number of studies, few studies were available according to tea subtypes, study populations from Europe and US, and genders, which have led to unstable results in or restriction to secondary analysis. Finally, our conclusions are based on results from observational studies. Especially, as the number of available cohort studies was limited, we also included cross-sectional studies in our meta-analysis to increase the statistical power. Thus, a causal association between tea consumption and cognitive disorders cannot be determined with the present data alone.

In conclusion, the results of our meta-analysis, involving 17 independent observational studies, provide significant evidence of an inverse and a linear relationship of tea consumption with the occurrence of cognitive disorders. A greater increment of tea drinking led to a greater magnitude in risk reduction. Further well-designed long-term randomized controlled trials (RCTs) are needed to confirm our findings.

## MATERIALS AND METHODS

### Search strategy and eligibility criteria

The guidelines published by the Meta-analysis of Observational Studies in Epidemiology (MOOSE) group was followed to complete the meta-analysis (Supplementary Table 1) [73]. Eligible studies that published in the international journals were searched in electronic databases of Pubmed, Embase, and Cochrane Library (from 1965 to Jan 19, 2017) by two investigators. We used the following MeSH and free-text terms in the search strategy: “Tea [Mesh]”, “Cognitive Dysfunction [Mesh]”, “Alzheimer Disease [Mesh]”, “Dementia [Mesh]”, “tea consumption”, “tea intake”, “tea”, “cognitive decline”, “cognitive impairment”, “cognitive disorder”, “dementia”, “Alzheimer disease”, and “Alzheimer's disease”. The search was restricted to studies in human beings and publications in English language. The reference lists of identified articles and relevant reviews were also checked for potentially eligible studies.

Studies that met the following criteria were included in our meta-analysis: (1) examination of tea consumption as the variable of interest; (2) determination of incidence of cognitive impairment, cognitive decline, dementia, or Alzheimer's disease as the outcome of interest; and (3) reporting the relative risks (RR) or odds ratios (OR) of cognitive disorders, and 95% confidence intervals (CI), or sufficient data with which to calculate these, according to the different levels of tea consumption. Studies about animal experiment, mechanistic research and review research were excluded.

### Data extraction and study quality evaluation

The following data from each included study were extracted by two investigators: first author, publication year, country, study design, sample size, mean age of the participants, follow-up duration of cohort studies, disease type of the outcome (cognitive impairment, cognitive decline, dementia, or Alzheimer's disease), exposure variable (tea type), exposure variable ascertainment method, disease ascertainment methods, categories of tea consumption, risk estimates with CIs, and confounding factors adjusted for. We included the most adjusted estimate when a study reported more than one risk estimate. Two investigators assessed the quality of each study, using the Newcastle-Ottawa Scale (NOS) recommended by Wells and colleagues [74]. The NOS score of each included study ranges from 1 to 9 stars for cohort and case-control studies and 1 to 5 stars for cross-sectional studies.

### Statistical analyses

We performed meta-analyses of risk estimates for cognitive disorders comparing the highest category of exposure to tea consumption with the lowest category. The results of studies using cognitive impairment, cognitive decline, dementia and Alzheimer's disease as the outcomes are presented separately. If a single study reported results for different populations (men and women), different types of tea consumption (green tea and black tea) or different assessment methods of cognitive disorders but did not report the overall results, the results for each population, type of tea consumption and assessment method of cognitive disorders were calculated as a different study [75]. We first conducted a meta-analysis of all types of tea consumption and the risk of cognitive disorders, and then we conducted meta-analyses for this association in different sexes and types of tea consumption, respectively. Subsequently, we conducted a dose-response meta-analysis from the correlated natural log of ORs across categories of tea consumption [76, 77], to estimate linear trends of OR for cognitive disorders per 100, 300, and 500 ml/day increment in tea consumption. As studies reported results for tea consumption in cups, we derived milliliter by assuming that the average cup equals to 215 ml [32]. We converted the level of consumption category based on the calculated midpoint of tea consumption if the study did not report the median of exposure category. If the maximum dose was fixed unlimitedly (e.g. ≥2 cups/d), we assumed that the mean was 25% larger than the lower level of the specific category [78]. Supplementary Table 2 summaries the definition of tea consumption and the means of conversion of categories within each study.

We used a random effects model to estimate the pooled ORs and 95% CIs to take into account the heterogeneities between studies. χ^2^ test and *I*^2^ statistic were used to evaluate the heterogeneity between studies. Sensitivity analysis was performed for the main meta-analysis by removing each individual study from the meta-analysis [79]. We visually assessed publication bias for the main meta-analysis by using funnel plots. Then we used Egger's regression test [80] and Begg-Mazumdar test [81] to further assess publication bias. All statistical analyses were performed using Stata Version 12.0 software (Stata Corp, College Station, TX).

## SUPPLEMENTARY FIGURES AND TABLES




