# Supplementary material for: Association between tea consumption and risk of cognitive disorders: A dose-response meta-analysis of observational studies
Source: Oncotarget. 2017 Apr 26;8(26):43306–21. doi: 10.18632/oncotarget.17429 (PMC5522147; doi:10.18632/oncotarget.17429)
Supplement: Supplementary file 1 [file oncotarget-08-43306-s001.pdf]

Association between tea consumption and risk of cognitive disorders: A dose-response meta-analysis of observational studies

SUPPLEMENTARY FIGURES AND TABLES

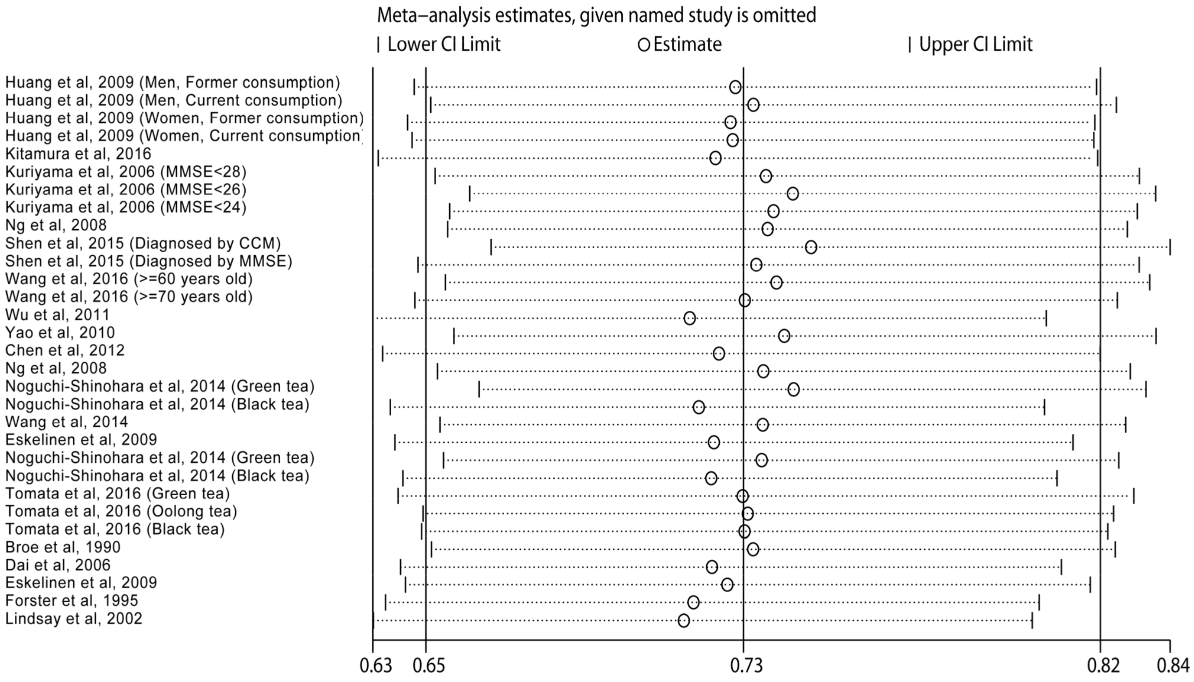

Supplementary Figure 1: Sensitivity analysis. Relative risk of cognitive disorders according to the highest vs. lowest category of tea consumption by omitting one study in turn.

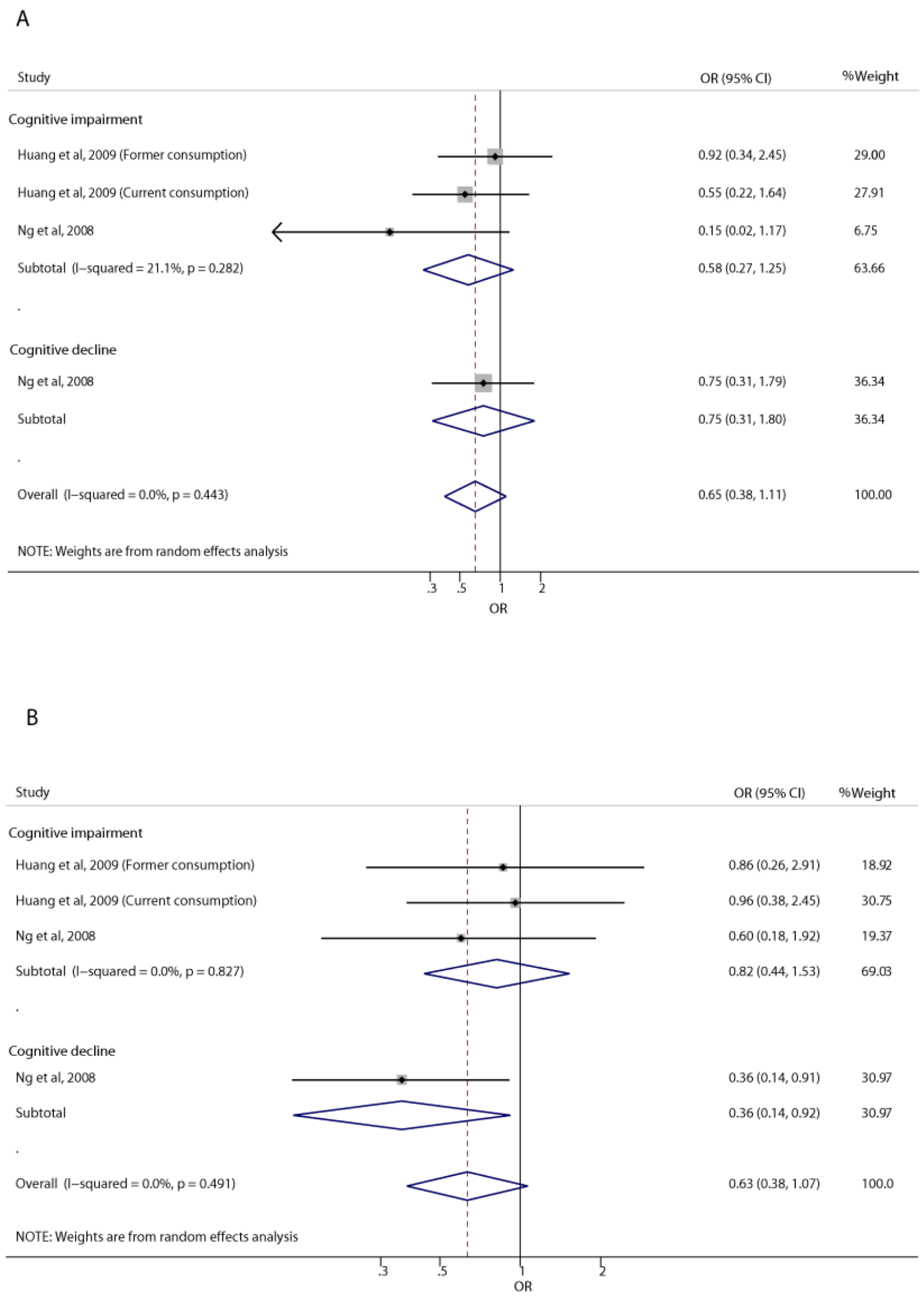

Supplementary Figure 2: Relative risks of cognitive disorders according to the highest vs. lowest category of tea consumption among men **(A)** and women **(B)**.

Supplementary Table 1: MOOSE Checklist

See Supplementary File 1

Supplementary Table 2: Levels of tea consumption and the conversions of categories

Reference	Exposure variable	Original categories	Conversional categories [a]
Dai et al, 2006	Tea of all types	<1 cup/wk	15.4 ml/day
		1-2 cups/wk	46.1 ml/day
		>=3 cups/wk	115.2 ml/day
Kitamura et al, 2016	Green tea	None	0
		1-6 cups/wk	107.5 ml/day
		1 cup/d	215 ml/day
Kuriyama et al, 2006	Green tea	<=3 cups/wk	46.1 ml/day
		4-6 cups/wk or 1 cup/d	153.6 ml/day
		>=2 cups/d	537.5 ml/day
Ng et al, 2008	Tea of all types	<1 cup/wk	15.4 ml/day
		1-3 cups/wk	61.4 ml/day
		>3 cups/wk but <3 cups/d	368.6 ml/day
		>=3 cups/d	806.3 ml/day
Noguchi-Shinohara et al, 2014	Green tea	None	0 ml/day
		1-6 cups/week	107.5 ml/day
		1 cup/day	215 ml/day
Shen et al, 2015	Tea of all types	Non-consumption	0 ml/day
		<2 cups/d	215 ml/day
		2-4 cups/d	645 ml/day
		>=4 cups/d	1075 ml/day
Tomata et al, 2016	Green tea	<1 cup/day	107.5 ml/day
	Black tea	1-2 cups/day	322.5 ml/day
	Oolong tea	3-4 cups/day	752.5 ml/day
		>=5 cups/day	1343.75 ml/day
Wu et al, 2011	Tea of all types	No	0
		<1 cup/wk	15.4 ml/day
		>=1 cup/wk	38.4 ml/day

[a] We assume that one cup of tea is 215 ml. We converted the level of consumption category based on the calculated midpoint of tea consumption if the study did not report the median of exposure category. If the maximum dose was fixed unlimitedly (e.g. >=2 cups/d), we assumed that the mean was 25% larger than the lower level of the specific category.

Supplementary Table 3: Quality assessment of the included studies (cohort studies)

See Supplementary File 1

Supplementary Table 4: Quality assessment of the included studies (case-control studies)

Study	Selection				Comparability	Exposure			Overall quality assessment score (of a maximum of 9)
	Is the case definition adequate?	Representativeness of the cases	Selection of controls	Definition of Controls	Comparability of cases and controls on the basis of the design or analysis	Ascertainment of exposure	Same method of ascertainment for cases and controls	Non-Response rate	
Broe et al, 1990	* Yes, with independent validation	* Somewhat representative of the average population in the community	* Drawn from the same community as the cases	* No history of disease	* The study controls for age, sex and the general practice of origin	* Secure record	* Yes	* Same rate for both groups	8
Chen et al, 2012	* Yes, with independent validation	* Truly representative of the average population in the community	* Drawn from the same community as the cases	* No history of disease	The study did not control for other factors	* Secure record	* Yes	* Same rate for both groups	7
Forster et al, 1995	* Yes, with independent validation	* Somewhat representative of the average population in the community	* Drawn from the same community as the cases	* No history of disease	The study did not control for other factors	* Secure record	* Yes	* Same rate for both groups	7

Supplementary Table 5: Quality assessment of the included studies (cross-sectional studies)

See Supplementary File 1
