# Supplementary material for: Association between tea consumption and risk of cognitive disorders: A dose-response meta-analysis of observational studies
Source: Oncotarget. 2017 Apr 26;8(26):43306–21. doi: 10.18632/oncotarget.17429 (PMC5522147; doi:10.18632/oncotarget.17429)
Supplement: Supplementary file 2 [file oncotarget-08-43306-s002.doc]

**Table S1: MOOSE Checklist**

| **Criteria** | | **Brief description of how the criteria were handled in the meta-analysis** |
| --- | --- | --- |
| **Reporting of background should include** | |  |
| √ | Problem definition | Previous studies find a favorable relation between tea consumption and risk of cognitive disorders, although sometimes the results are inconsistent. Moreover, the strength of the favorable relation remains uncertain due to the differences in participants and methodological methods used in the previous studies. |
| √ | Hypothesis statement | Tea consumption is inversely associated with the risk of cognitive disorders. |
| √ | Description of study outcomes | Cognitive disorders |
| √ | Type of exposure or intervention used | Tea consumption |
| √ | Type of study designs used | Observational study |
| √ | Study population | Elderly with cognitive disorders and controls |
| **Reporting of search strategy should include** | |  |
| √ | Qualifications of searchers (eg, librarians and investigators) | The credentials of the two investigators are provided in the author list. |
| √ | Search strategy, including time period included in the synthesis and keywords | Pubmed, Embase, and Cochrane Library (from 1965 to Jan 19, 2017).  Keywords: “Tea [Mesh]”, “Cognitive Dysfunction [Mesh]”, “Alzheimer Disease [Mesh]”, “Dementia [Mesh]”, “tea consumption”, “tea intake”, “tea”, “cognitive decline”, “cognitive impairment”, “cognitive disorder”, “dementia”, “Alzheimer disease”, and “Alzheimer’s disease”. |
| √ | Effort to include all available studies, including contact with authors | The references of all retrieved articles and recent reviews were also manually reviewed. |
| √ | Databases and registries searched | Pubmed, Embase, and Cochrane Library |
| √ | Search software used, name and version, including special features used (eg, explosion) | We did not employ a special search software. |
| √ | Use of hand searching (eg, reference lists of obtained articles) | References of all retrieved articles and recent reviews were reviewed. |
| √ | List of citations located and those excluded, including justification | Details of the literature search process are outlined in the flow chart. |
| √ | Method of addressing articles published in languages other than English | We placed restrictions on English. |
| √ | Method of handling abstracts and unpublished studies | No attempt was made to find articles in languages other than English or to contact authors of unpublished works. |
|  | Description of any contact with authors. | - |
| **Reporting of methods should include** | |  |
| √ | Description of relevance or appropriateness of studies assembled for assessing the hypothesis to be tested | The inclusion criteria are presented in the “Search strategy and eligibility criteria” section. |
| √ | Rationale for the selection and coding of data (eg, sound clinical principles or convenience) | Two researchers independently extracted the following data from each publication: first author, publication year, country, study design, sample size, mean age of the participants, follow-up duration of cohort studies, disease type of the outcome (cognitive impairment, cognitive decline, dementia, or Alzheimer’s disease), exposure variable (tea type), exposure variable ascertainment method, disease ascertainment methods, categories of tea consumption, risk estimates with CIs, and confounding factors adjusted for. |
| √ | Documentation of how data were classified and coded (eg, multiple raters, blinding, and inrerrater reliability) | Data were independently extracted and analyzed by two investigators and final decision was reached by consensus. |
| √ | Assessment of confounding (eg, comparability of cases and controls in studies where appropriate) | Table 1 presents the adjustment factors for each study. |
| √ | Assessment of study quality, including blinding of quality assessors; stratification or regression on possible predictiors of study results | The quality of each study was assessed by two investigators, using the Newcastle-Ottawa Scale. |
| √ | Assessment of heterogeneity | χ^2^ test and *I*^2^ statistic were used to explore the heterogeneity among studies. |
| √ | Description of statistical methods (eg, complete description of fixed or random effects models, justification of whether the chosen models account for predictors of study results, dose-response models, or cumulative meta-analysis) in sufficient detail to be replicated | Description of methods of meta-analyses, subgroup analyses, dose-response meta-analysis, sensitivity analysis, and assessment of publication bias are detailed in the “Statistical analysis” section. |
| √ | Provision of appropriate tables and graphics | One main table and five supplemental tables are provided. One flow chart and three forest plots appear in the main text. |
| **Reporting of results should include** | |  |
| √ | Graph summarizing individual study estimates and overall estimate | Figures 2, 3, 5 |
| √ | Table giving descriptive information for each study included | Table 1 |
| √ | Results of sensitivity testing (eg, subgroup analysis) | “Results” section; Figure S1 |
| √ | Indication of statistical uncertainty of findings | 95% confidence intervals were presented with all summary effect estimates. |
| **Reporting of discussion should include** | |  |
| √ | Quantitative assessment of bias (eg, publication bias) | “Results” section, and Figure 6. |
| √ | Justification for exclusion (eg, exclusion of non-English-language citations) | The details of the exclusion of studies are shown in Flow chart. |
| √ | Assessment of quality of included studies | Tables S3-S5. |
| **Reporting of conclusions should include** | |  |
| √ | Consideration of alternative explanations for observed results | We discussed that the meta-analysis is based on observational studies, thus, we cannot exclude chance, residual or unmeasured confounding as alternative explanation for our findings. |
| √ | Generalization of the conclusions (ie, appropriate for the data presented and within the domain of the literature review) | We discussed that the results of current study support the concept that tea consumption is associated with reduced risk of cognitive disorders, however, it does not establish a causal relation |
| √ | Guidelines for future research | We discussed that Further well-designed long-term randomized controlled trials (RCTs) are needed to confirm our findings. |
| √ | Disclosure of funding source | The funding information is shown in the text. |

**Table S3: Quality assessment of the included studies (cohort studies)**

| Study | Selection | | | | Comparability | Outcome | | | Overall quality assessment score (of a maximum of 9) |
| --- | --- | --- | --- | --- | --- | --- | --- | --- | --- |
|  | Representativeness of the exposed cohort | Selection of the non exposed cohort | Ascertainment of exposure | Demonstration that outcome of interest was not present at start of study | Comparability of cohorts on the basis of the design or analysis | Assessment of outcome | Was follow-up long enough for outcomes to occur | Adequacy of follow up of cohorts |  |
| Dai et al, 2006 | *Truly representative of the average population in the community | * Drawn from the same community as the exposed cohort | * Structured interview | The study didn’t demonstrate that dementia was not present at start of study | ** Study controls for years of education, gender, regular physical activity, BMI, baseline CASI score, olfaction diagnostic group, total energy intake, intake of saturated, monounsaturated, and polyunsaturated fatty acids, ApoE genotype, smoking status, alcohol drinking, supplementation of vitamin C, vitamin E, and multivitamin, tea drinking, fruit and vegetable juice drinking, dietary intake of vitamin C, vitamin E, and β-carotene. | * Independent blind assessment | * The study selected an adequate follow up period for outcome of interest | Subjects lost to follow up might introduce bias (52%) follow up | 7 |
| Eskelinen et al, 2009 | * Truly representative of the average population in the community | * Drawn from the same community as the exposed cohort | * Structured interview | The study didn’t demonstrate that dementia was not present at start of study | ** Study controls for age, sex, education, follow-up time, community of residence, midlife smoking, systolic blood pressure, serum total cholesterol, body mass index, and physical activity | * Independent blind assessment | * The study selected an adequate follow up period for outcome of interest | *Subjects lost to follow up might not introduce bias (100% follow up) | 8 |
| Lindsay et al, 2002 | * Truly representative of the average population in the community | * Drawn from the same community as the exposed cohort | * Structured interview | * The study demonstrated that dementia was not present at start of study | * Study controls for age, sex, and education | * Independent blind assessment | * The study selected an adequate follow up period for outcome of interest | Subjects lost to follow up might introduce bias (45%) follow up | 7 |
| Noguchi-Shinohara et al, 2014 | * Somewhat representative of the average population in the community | * Drawn from the same community as the exposed cohort | * Structured interview | * The study demonstrated that dementia was not present at start of study | ** Study controls for age, sex, history of hypertension, diabetes mellitus, typerlipidemia, education, APOE ε4 carrier status, alcohol drinking, smoking, physical activities and/or hobbies, and coffee consumption | * Independent blind assessment | * The study selected an adequate follow up period for outcome of interest | Subjects lost to follow up might introduce bias (68%) follow up | 8 |
| Tomata et al, 2016 | * Truly representative of the average population in the community | * Drawn from the same community as the exposed cohort | * Structured interview | * The study demonstrated that dementia was not present at start of study | * Study controls for age, sex, history of disease, educational level, smoking, alcohol drinking, BMI, psychological distress score, time spent walking, social support, participation in community activities, motor function score, consumption volume of specific foods coffee consumption, and energy intake | * Independent blind assessment | * The study selected an adequate follow up period for outcome of interest | *Subjects lost to follow up might not introduce bias (100%) follow up | 9 |
| Wang et al, 2014 | * Somewhat representative of the average population in the community | * Drawn from the same community as the exposed cohort | * Structured interview | * The study demonstrated that dementia was not present at start of study | * Study controls for age and gender | * Independent blind assessment | The study did not select an adequate follow up period for outcome of interest | *Subjects lost to follow up might not introduce bias (100% follow up) | 7 |

**Table S5: Quality assessment of the included studies (cross-sectional studies)**

| Study | Selection | | Comparability | Exposure | Overall quality assessment score (of a maximum of 5) |
| --- | --- | --- | --- | --- | --- |
|  | Representativeness of the sample | Ascertainment of exposure | Comparability of groups on the basis of the design or analysis | Assessment of outcome |  |
| Huang et al, 2009 | * Truly representative of the average population in the community | * Structured interview | * * Study controls for age, sex, sleep habits, educational level, religion habits, and temperament | * Independent blind assessment | 5 |
| Kitamura et al, 2016 | * Somewhat representative of the average population in the community | * Structured interview | * * Study controls for age, BMI, history of stroke, history of myocardial infarction, walking time, alcohol intake, and fruit consumption | * Independent blind assessment | 5 |
| Kuriyama et al, 2006 | * Truly representative of the average population in the community | * Structured interview | * * Study controls for age, sex, energy intake, intake of nondietary vitamin C or E, fish consumption, green or yellow vegetable consumption, mild leisure-time physical activity, vigorous leisure-time physical activity, smoking, and alcohol use | * Independent blind assessment | 5 |
| Ng et al, 2008 | * Truly representative of the average population in the community | * Structured interview | * * Study controls for age, sex, education, smoking, alcohol consumption, BMI, hypertension, diabetes, heart disease, stroke, depression, APOEε4, physical activities, social and productive activities, vegetable and fruit consumption, fish consumption, and coffee consumption | * Independent blind assessment | 5 |
| Shen et al, 2015 | * Truly representative of the average population in the community | * Structured interview | * * Study controls for age, sex, race, education, marriage, tea concentration, tea categories, physical examinations, family status, disease situation, behavioral risk factors, dietary intake, nutrition supplement, depression and ADL | * Independent blind assessment | 5 |
| Wang et al, 2016 | * Truly representative of the average population in the community | Not mention | * Study does not mention what factors control for. | * Independent blind assessment | 3 |
| Wu et al, 2011 | * Truly representative of the average population in the community | * Structured interview | * * Study controls for age, gender, educational level, marital status, social support, hyperlipidemia, stroke, physical function, depressive symptoms, self-rated health, cigarette smoking, leisure-time physical activity, fruits and vegetables consumption, coffee intake, multivitamin intake, and BMI | * Independent blind assessment | 5 |
| Yao et al, 2010 | * Truly representative of the average population in the community | * Structured interview | Study did not control for other factors | * Independent blind assessment | 3 |
